# Signatures of positive selection in African Butana and Kenana dairy zebu cattle

**DOI:** 10.1371/journal.pone.0190446

**Published:** 2018-01-04

**Authors:** Hussain Bahbahani, Bashir Salim, Faisal Almathen, Fahad Al Enezi, Joram M. Mwacharo, Olivier Hanotte

**Affiliations:** 1 Department of Biological Sciences, Faculty of Science, Kuwait University, Kuwait city, Kuwait; 2 Department of Parasitology, Faculty of Veterinary Medicine, University of Khartoum Khartoum North, Sudan; 3 Department of Veterinary Public Health and Animal Husbandry, College of Veterinary Medicine, King Faisal University, Al-Hasa, Kingdom of Saudi Arabia; 4 Small Ruminant Genomics Group, International Centre for Agricultural Research in the Dry Areas (ICARDA), Addis Ababa, Ethiopia; 5 Cells, Organisms and Molecular Genetics, School of Life Sciences, University of Nottingham, Nottingham, United Kingdom; 6 LiveGene, International Livestock Research Institute (ILRI), Addis Ababa, Ethiopia; Institute of Animal Sciences, GERMANY

## Abstract

Butana and Kenana are two types of zebu cattle found in Sudan. They are unique amongst African indigenous zebu cattle because of their high milk production. Aiming to understand their genome structure, we genotyped 25 individuals from each breed using the Illumina BovineHD Genotyping BeadChip. Genetic structure analysis shows that both breeds have an admixed genome composed of an even proportion of indicine (0.75 ± 0.03 in Butana, 0.76 ± 0.006 in Kenana) and taurine (0.23 ± 0.009 in Butana, 0.24 ± 0.006 in Kenana) ancestries. We also observe a proportion of 0.02 to 0.12 of European taurine ancestry in ten individuals of Butana that were sampled from cattle herds in Tamboul area suggesting local crossbreeding with exotic breeds. Signatures of selection analyses (*iHS* and *Rsb*) reveal 87 and 61 candidate positive selection regions in Butana and Kenana, respectively. These regions span genes and quantitative trait loci (QTL) associated with biological pathways that are important for adaptation to marginal environments (e.g., immunity, reproduction and heat tolerance). Trypanotolerance QTL are intersecting candidate regions in Kenana cattle indicating selection pressure acting on them, which might be associated with an unexplored level of trypanotolerance in this cattle breed. Several dairy traits QTL are overlapping the identified candidate regions in these two zebu cattle breeds. Our findings underline the potential to improve dairy production in the semi-arid pastoral areas of Africa through breeding improvement strategy of indigenous local breeds.

## Introduction

The history of African cattle started ~5000 BC with the initial migration of taurine (*Bos taurus taurus*) cattle to the continent from their center of domestication in the Near East [1, 2]. This was followed by the introduction of indicine (*Bos taurus indicus* (zebu)) cattle from their centre of domestication in the Indus valley [3]. It is widely accepted that the zebu cattle introduction to Africa took place in two waves, ~2000 BC and 700 AD [4]. The later was considered to be the major event that led to the genetic introgression with the native African taurine cattle. Indeed, all African cattle analyzed to date carry taurine mtDNA [5–7] indicating a male-mediated zebu introgression. This introgression lead to African cattle populations with zebu phenotypes and an admixed genome composed of both African taurine and zebu ancestries [4, 8, 9].

More than 150 cattle breeds are found in Africa with an estimated population size of about 312 million heads (FAOSTAT 2014; accessed May 02 2017; http://www.fao.org/faostat/en/#data/QA). The estimated cattle population size in Sudan is about 44 million heads (Annual report of the Federal Ministry of Animal Resources, Fisheries and Ranges, 2016). These are mainly of zebu type commonly referred to as Kenana, Butana, Baggara and Nilotic cattle [10].

Butana and Kenana cattle are classified as Large East African zebu cattle [11]. Butana cattle are kept mainly by nomadic pastoralists (Batahin and Shukria ethnic groups) and agro-pastoralists (Dongola and Shendi ethnic groups). The Kenana cattle have remained largely under the guardianship of semi-nomadic pastoralists (Fung and White Nile ethnic groups).

The Butana cattle, also referred to as Dar El Reih cattle across the White Nile in the northern part of Darfur and Kordofan, inhabit the Butana plains in central Sudan between the Blue Nile and Atbara rivers. The Butana plain receives around 300 mm of rainfall annually and has an eight months dry season. The traditional habitat of the Kenana cattle is the Blue Nile province, which is to the east of the confluence of the Blue and White Niles at Khartoum, and south-east to the Ethiopian border as well as south of Khartoum. The area receives 300–800 mm of rainfall annually with a six months dry season. Some irrigation agricultural schemes, e.g. the Gezira scheme, are found in the area [11, 12]. Various infectious diseases are prevalent in Butana and Kenana habitats, such as theileriosis, foot and mouth disease, brucellosis and trypanosomosis [12, 13].

Phenotypically, the Butana cattle have deep-red coat color while the Kenana spot brown-red coats as calves which turns to grey between three and six months of age [14]. With a milk production performance approximating that of their counterparts from the Indian sub-continent, *viz* Sahiwal, Red Sindhi, Radhi, Tharparkar, Hariana, Kankrej and Gir, the Butana and Kenana cattle are considered to be African indigenous zebu types of dairy cattle [11]. Under farmer management they produce, on average, 538.26 and 598.73 Kg of milk per lactation, respectively [12]. However, in research stations Butana cattle produce about 1662 kg of milk per lactation [15], whilst Kenana cattle produce between 1400 to 2100 kg of milk per lactation [14]. The average length of lactation for the Butana is 268.17 days [15] while that of Kenana ranges between 198 to 257 days [14]. Although these two cattle breeds show high potential dairy production, genetic studies to improve their productivity are scarce. Recently, a study in Butana cattle identified haplotypes in the milk protein casein genes cluster on BTA 6, which might be associated with milk protein percentage [16].

The investigation of genome-wide signatures of selection for production or adaptive traits have now been undertaken for several cattle breeds using either genome-wide single nucleotide polymorphism (SNP) and/or full genome sequence data, e.g., [17–20]. Candidate regions with signatures of positive selection have been identified in the genomes of commercial breeds, such as Holstein, Angus, Charolais and Fleckvieh [20–22]. These sweep regions span genes, e.g. *DGAT1* and *GHR* [22, 23], and quantitative trait loci (QTL) associated with productivity traits, such as milk yield and composition [21], and muscle development gene (*MSTN*) [22]. The genomes of indigenous cattle populations from tropical regions have also been characterized (e.g. East African Shorthorn Zebu [18, 19], West and East African [24, 25] and the Caribbean (Creole) cattle [26]), for candidate signatures of positive selection using genome-wide SNP chips as well as full genome sequence data. These regions span genes associated with different biological traits, such as immunity, reproduction and heat tolerance. Unlike the commercial breeds, whose phenotypic and production traits are mainly influenced by human-mediated selection, natural rather than human selection seems to have shaped the genome of these indigenous cattle.

In this study, we assessed the genomic profile of two indigenous populations of East African zebu-type dairy cattle, Butana and Kenana, using genotype data generated using the BovineHD Genotyping BeadChip [27]. We explored their genomes for signatures of positive selection using intra- and inter-population approaches. As observed in other African cattle populations, our analysis revealed the genomes of the two populations to be an admixture of indicine and taurine ancestries. We also identified several candidate selection sweep regions that spanned genes associated with different biological pathways, such as reproduction, heat stress and coat color, and QTL linked to different milk-production traits and trypanotolerance. These results may help in improving the designing of breeding programs in indigenous African cattle breeds and in particular in crossbreeding or within-breed selection programmes for the Butana and Kenana breeds.

## Material and methods

### Studied samples, SNP genotyping and quality control

Genomic DNA was extracted from whole blood, spotted on FTA^®^ cards (Whatman Inc., New Jersey, USA), from 25 samples each of Butana (BUT) and Kenana (KEN) using an in-house protocol. Standard techniques were used to collect blood samples. The procedure was reviewed and approved by the University of Khartoum, Sudan. Informed consent was sought from animal owners and research stations. Eleven samples of Butana were collected from Atbara Livestock Research Station, while 14 were sampled from farmer’s herds in Tamboul area. The Kenana samples were collected from farmers herds in Rabak area “S1 Table and S1 Fig”. All the samples were genotyped for 786,799 SNPs using the Illumina BovineHD Genotyping BeadChip [27]. SNP genotype data generated using the same chip were included in this study from 92 non-European introgressed small East African shorthorn zebu (EASZ) from [19] and 59 Holstein-Friesian (HOL), 32 Jersey (JER), 24 N’Dama (NDM), 35 Nelore (NEL) and 18 Sheko (SHK) cattle were provided by Dr Tad Sonstegard (USDA-ARS, Maryland) and previously described in [19].

Quality control (QC) filtering was conducted using the *check*.*marker* function of the GenABEL package [28] for R version 2.15.1 [29] on 741,959 autosomal SNPs with known mapping positions on the UMD3.1 bovine reference genome [30] that did not conflict with those on the Btau4.2 genome assembly (957 SNPs). Minor allele frequency (MAF) of less than 1% and SNP genotyping call rate of 95% were set as two filtering criteria that resulted in pruning out 24,424 and 22,099 SNPs, respectively. These included 8,465 SNPs that failed both criteria, leaving 703,901 SNPs for downstream analyses. Low genotyping call rate (< 95%) and high identity by state (IBS ≥ 95%) were also set as two filtering criteria. Two Nelore samples failed the IBS criteria and the one with the lower genotyping call rate was excluded from analysis.

### Analysis of genetic relationships and structure

Principal component analysis (PCA) and admixture analysis were conducted to assess the within as well as the between population genetic differentiation and admixture. PCA was performed for all the samples dataset, as well as for Butana and Kenana only. The *prcomp* function implemented in GenABEL package for R version 2.15.1 was used to perform the PCA.

Admixture analysis using ADMIXTURE 1.23 software [31] with cross-validation and 200 bootstraps for (1 ≤ *K* ≤ 8) was conducted on the whole dataset to determine the European taurine, Asian zebu and African taurine ancestries at genome-wide level and for each autosome separately. The optimal number of clusters was determined following [32] by calculating *Delta K* (*ΔK*) for each *K* value. The output files were graphically displayed by the *ggplot2* package [33] for R software.

### Analysis of signatures of positive selection

Two Extended Haplotype Homozygosity (EHH) based statistics, *Rsb* and integrated haplotype score (*iHS*), were used to assess genome-wide signatures of positive selection in Butana and Kenana cattle. Separate inter-population *Rsb* [34] analyses were performed between each of the Butana and Kenana cattle breeds and the European taurine (HOL and JER), EASZ, NDM, NEL and SHK cattle populations, respectively using the *rehh* package [35] of R version 2.15.1. The integrated site-specific EHH (EHHS) of each SNP for each population (*iES*) was calculated. The natural logarithm of the ratio between *iES*_*pop1*_ and *iES*_*pop2*_ was used to calculate the unstandardized *Rsb* values which were then standard-transformed based on the calculated median and standard deviation values. As the standardized *Rsb* values were normally distributed “S2A Fig”, one-tailed Z-tests were applied to identify statistically significant SNPs under positive selection in Butana or Kenana populations (positive *Rsb* values). One-sided *P*-values were derived as–log_10_(1-Φ(*Rsb*)), where Φ(*Rsb*) represents the Gaussian cumulative distribution function. A value of 4, equivalent to *P*-value = 0.0001, was used as a significant threshold.

Intra-population *iHS* analyses [36] were conducted separately for Butana and Kenana cattle populations, using the *rehh* package of R version 2.15.1. The integrated EHH of the reference and alternative alleles (*iHH*_*ref*_ and *iHH*_*alt*_) were calculated for each SNP with a within-population MAF ≥ 5%. The natural log of the ratio between *iHH*_*ref*_ and *iHH*_*alt*_ was used to derive the *iHS* values. As the standardized *iHS* values were also normally distributed “S2B Fig”, two-tailed Z-tests were applied to identify statistically significant SNPs under positive selection. Two-sided *P*-values were derived as–log_10_(1–2|ф(*iHS*)-0.5|), where Φ(*iHS*) represents the Gaussian cumulative distribution function. A value of 4, equivalent to *P*-value = 0.0001, was used to define the significant threshold. A candidate selection sweep region was defined if five consecutive SNPs with a maximum inter-marker distance of 500 kb passed the significance threshold. This inter-marker distance approximates the extent of linkage disequilibrium (LD) in different taurine and zebu cattle breeds [37] and has been used previously [19]. Moreover, beyond this genomic distance the mean pairwise LD statistic (r^2^) estimated using the *r2fast* function of the GenABEL package for the Butana and Kenana cattle drops below 0.1 “S3 Fig”.

For both EHH based statistics, haplotypes were constructed through phasing the genotyped SNPs using *fastPHASE* version 1.4 [38]. For this, the K10 and T10 criteria’s applied by [19, 39] were used.

### Functional characterization of the candidate regions

Candidate genes were considered if their boundaries fell within 25 kb from the most significant SNP in the candidate regions. A list of the protein-coding and RNA genes found within the candidate regions were also retrieved from the *Ensembl Genes 81* database, based on the UMD3.1 bovine reference genome, using the *BioMart* tool [40]. All the identified genes were processed using the functional annotation tool implemented in *DAVID* Bioinformatics resources 6.7 [41] to determine enriched functional terms. An enrichment score of 1.3, which is equivalent to the Fisher exact test *P*-value of 0.05, was used as a threshold to define the significantly enriched functional terms in comparison to the whole bovine reference genome background.

The bovine QTL that have been mapped on the bovine Btau 4.0 reference genome assembly were downloaded from the cattle QTL database (http://www.animalgenome.org/cgi-bin/QTLdb/BT/index). The QTL genome coordinates were then re-mapped on the bovine UMD 3.1 reference genome assembly using the NCBI genome remapping online service (https://www.ncbi.nlm.nih.gov/genome/tools/remap). The *intersectBed* function of the *BedTools* software [42] was used to overlap these QTL with the identified candidate regions.

## Results

### Genetic relationship between the cattle populations

**Fig 1:**
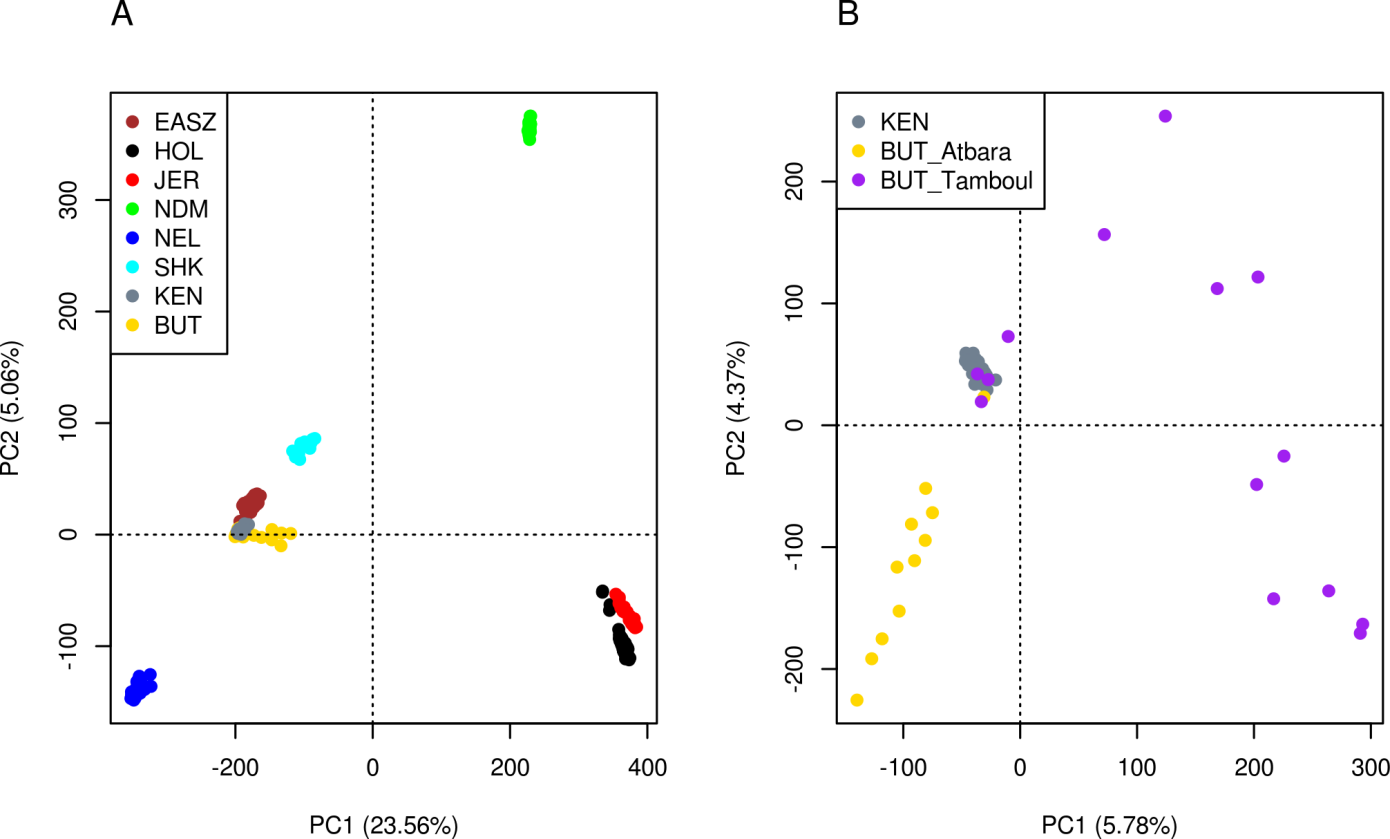
PCA plots showing the genetic relationship between cattle breeds. (A) all cattle breeds and (B) Butana and Kenana cattle.

The PCA and admixture analyses were used to assess genetic admixture and structure between the study populations and within the Butana and Kenana cattle. The first (PC1) and second (PC2) principal components reveals the previously described triangle-like 2-dimensional global organization of cattle genetic diversity “Fig 1A” [26]. The PC1, which explains 23.56% of the total variation, separates the indicine cattle from their taurine (African (NDM) and European (HOL, JER)) counterparts. The PC2, which explains 5.06% of the total variation, differentiates the African taurine (NDM) from the European taurine (JER, HOL). This PC also appears to separate the Asian indicine (NEL) from their African counterparts (EASZ, BUT, KEN). Generally, all the cattle found in East Africa (EASZ, SHK, KEN and BUT) are closely clustered together and occur at an intermediate position with respect to the location of the Asian zebu (NEL) and the African taurine (NDM) cattle. We also performed a separate PCA for Butana and Kenana cattle “Fig 1B”. Based on the clustering pattern, it reveals a lower level of genetic homogeneity in the Butana compared to the Kenana cattle. The latter clusters closely together while the former spreads out across the PCA plot with the highest variation being observed in Butana cattle from Tamboul, which are separated from the samples from Atbara Research station.

**Fig 2:**
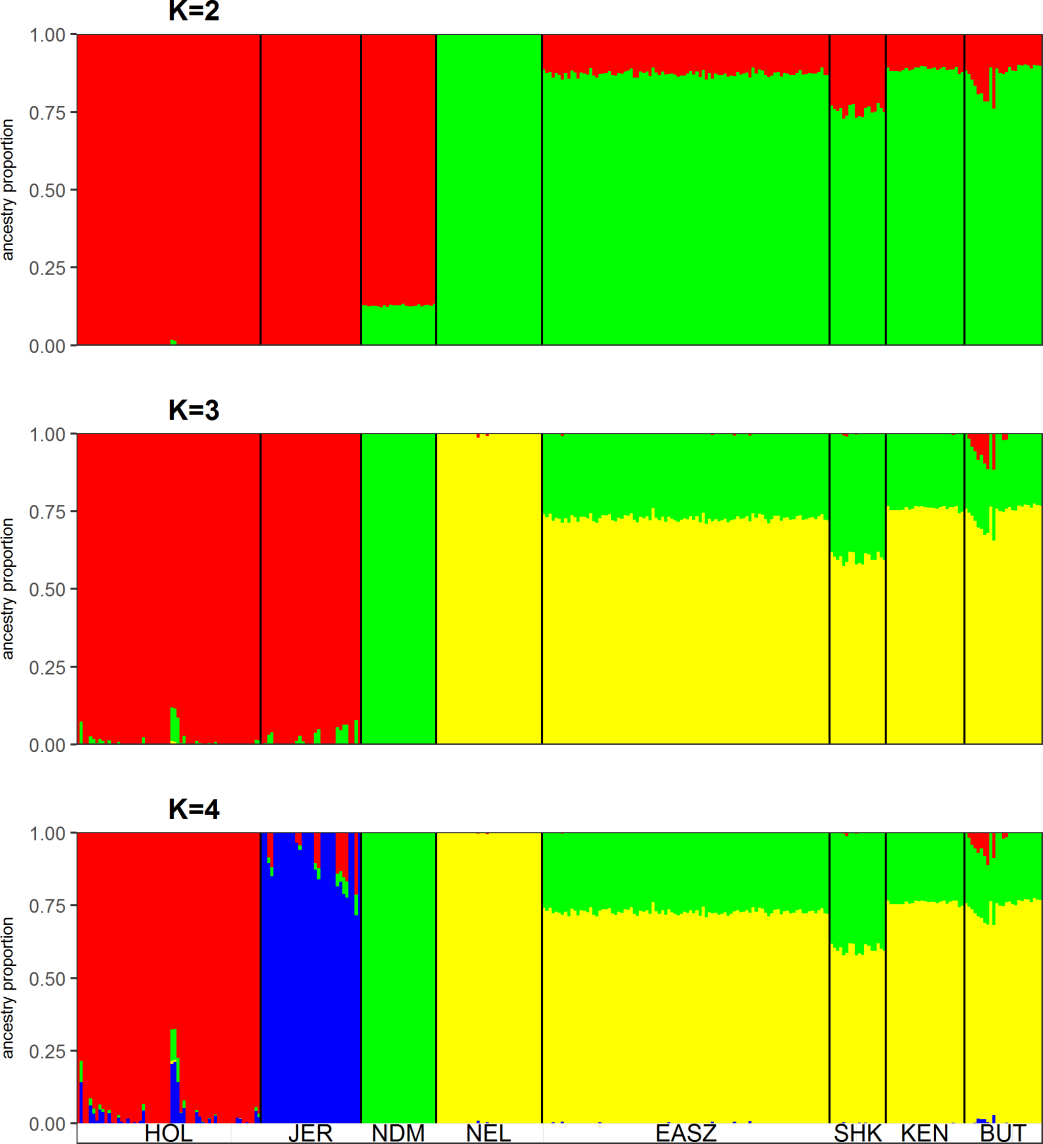
ADMIXTURE bar plots of genomic membership proportions from *K* = 2 to *K* = 4. Each sample is represented by a vertical line divided into *K* colours. HOL: Holstein, JER: Jersey, NDM: N’Dama, NEL: Nelore, SHK: Sheko, KEN: Kenana, BUT: Butana.

The *ΔK* approach indicates that the most optimal number of genetic clusters in the overall dataset is *K* = 2 “S4 Fig”. This level of clustering corresponds to the two ancestries defining the global cattle populations; indicine and taurine “Fig 2”. At *K* = 3, the African taurine differentiates from its European counterparts, with a minor level of shared background in Holstein-Friesian and Jersey animals “Fig 2”. Moreover, the genomes of Butana and Kenana are composed of indicine and taurine backgrounds “Fig 2”. The average proportions of these two backgrounds are in Butana 0.75 ± 0.03 and 0.23 ± 0.01, respectively. For Kenana, they are 0.76 ± 0.006 and 0.24 ± 0.006, respectively. European taurine genetic proportions, ranging from 0.02 to 0.12, can also be observed in ten Butana cattle sampled from farmers herds in Tamboul “Fig 2”.

At *K* = 4, the Jersey cattle separate from the Holstein-Friesian with a minor and variable level of common ancestry “Fig 2”. From *K* = 5 to *K* = 8, a separate African specific genome background can be identified in the cattle populations from East Africa (EASZ, SHK, KEN and BUT) as well as a substantial indicine ancestry and a lower level of taurine ancestry shared with the N’Dama cattle “S5 Fig”.

### Candidate regions under positive selection in Butana and Kenana cattle

**Fig 3:**
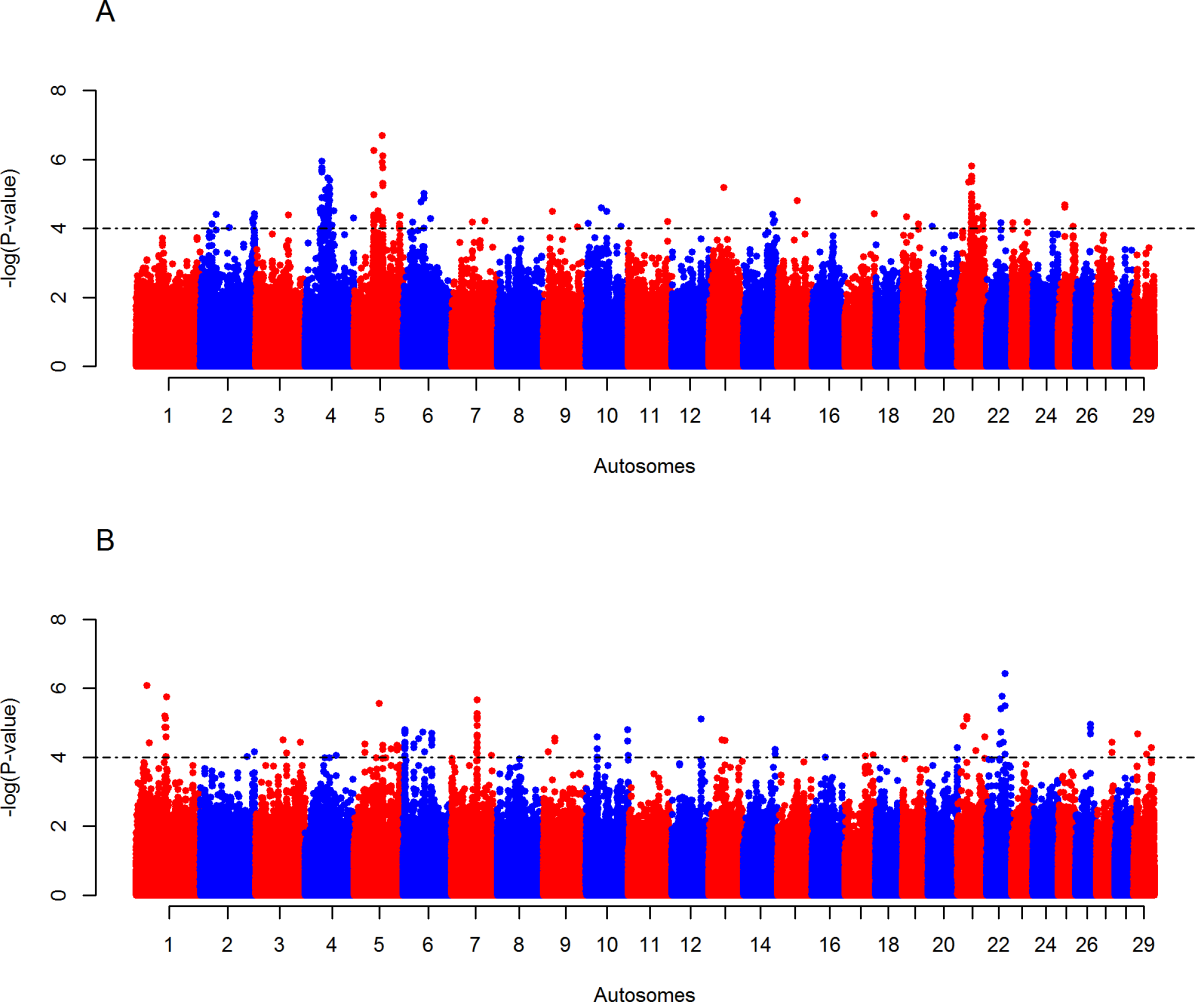
Manhattan plots of genome-wide *iHS* analyses on (A) Butana and (B) Kenana cattle. two-tailed Z-test is applied and the significance threshold is set at–log_10_ (two-tailed *P*-value) = 4.

**Fig 4:**
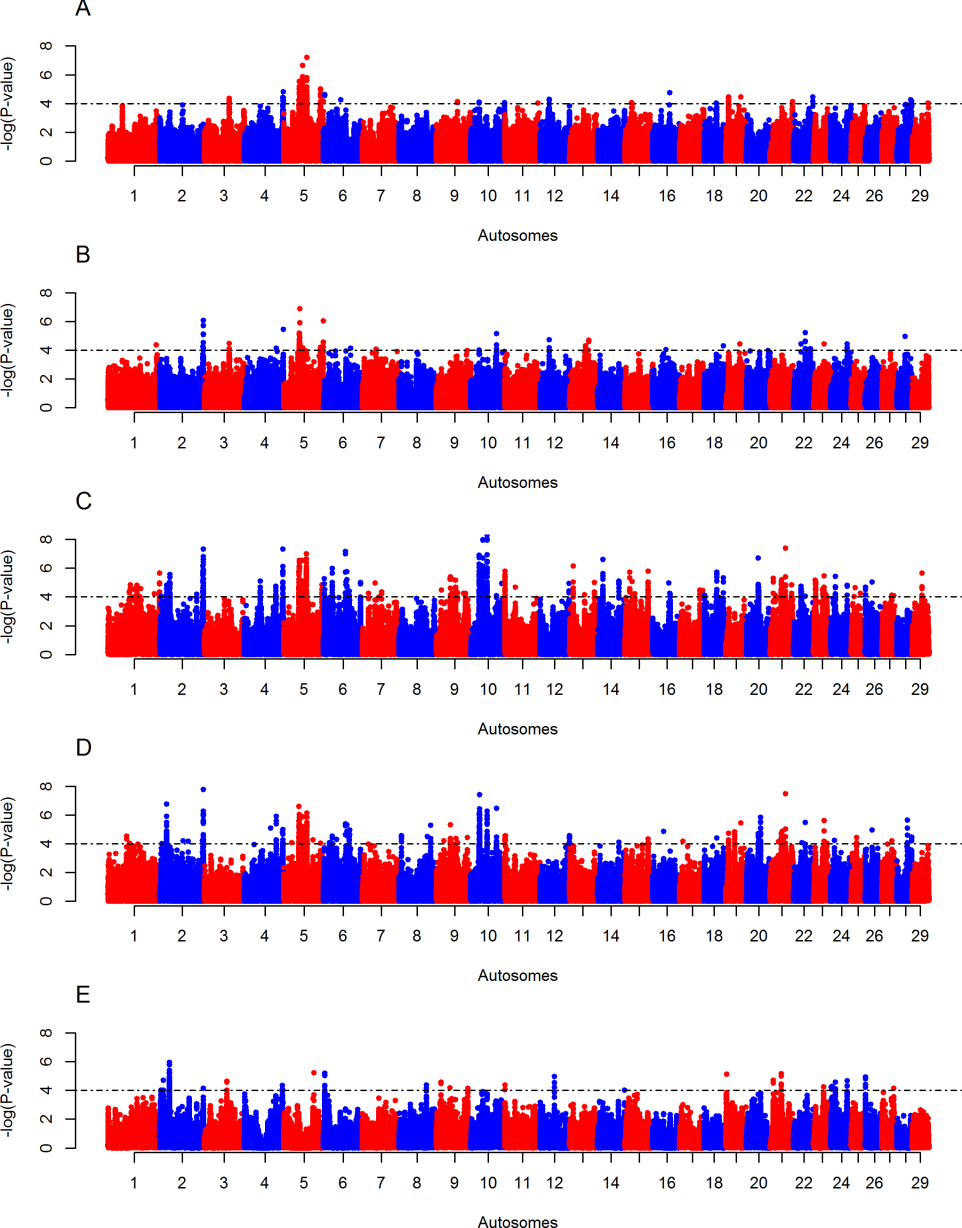
Manhattan plots of genome-wide autosomal *Rsb* analyses of Butana cattle. (A) European taurine (Holstein-Friesian and Jersey), (B) African taurine (N’Dama), (C) East African shorthorn zebu, (D) Sheko, and (E) Asian zebu (Nelore). One-tailed Z-test is applied and the significance threshold is set at–log_10_ (one-tailed *P*-value) = 4.

**Fig 5:**
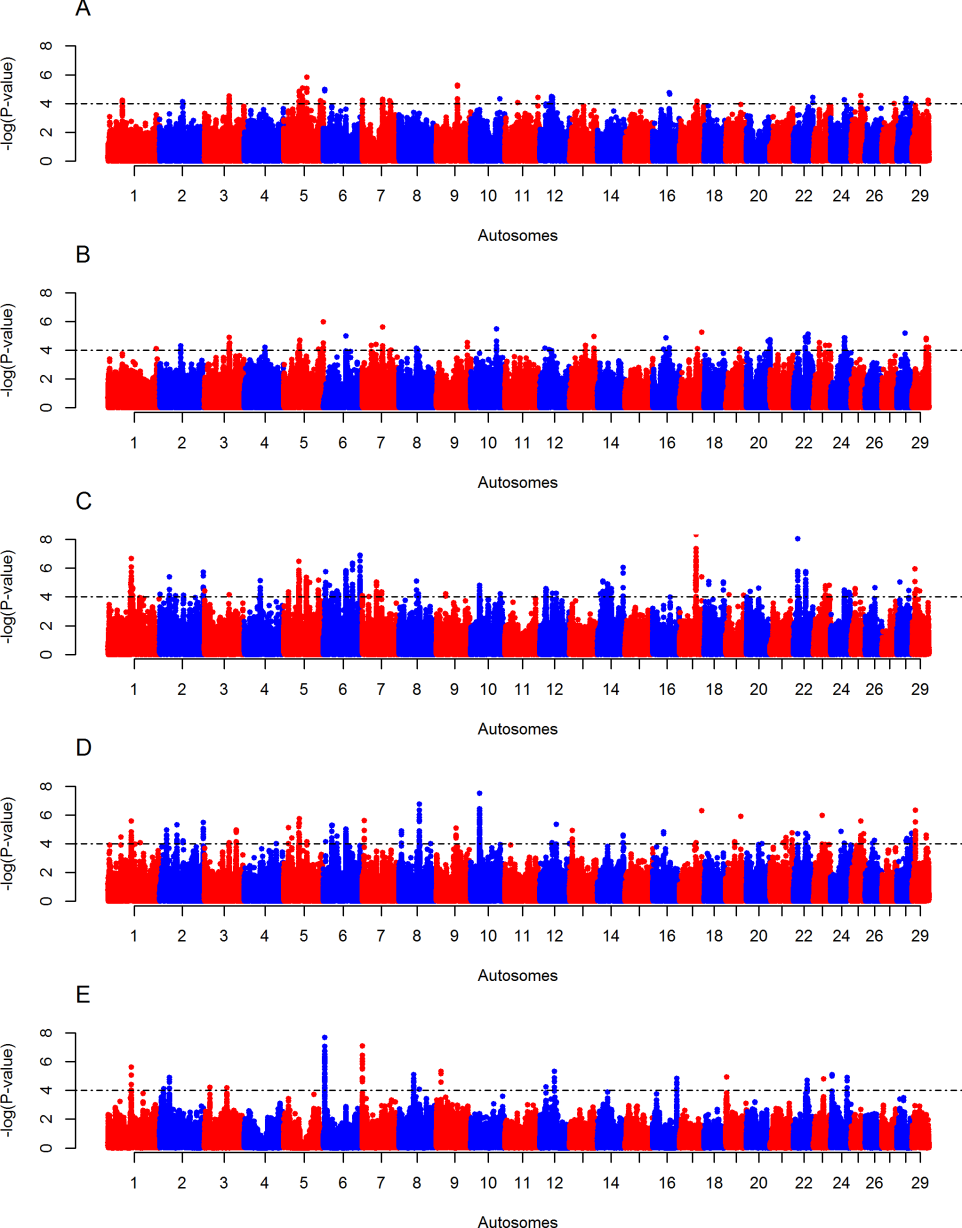
Manhattan plots of genome-wide autosomal *Rsb* analyses of Kenana cattle. (A) European taurine (Holstein-Friesian and Jersey), (B) African taurine (N’Dama), (C) East African shorthorn zebu, (D) Sheko and (E) Asian zebu (Nelore). One-tailed Z-test is applied and the significance threshold is set at–log_10_ (one-tailed *P*-value) = 4.

The intra-population *iHS* analyses reveal eight and two candidate regions in Butana and Kenana cattle, respectively “Fig 3 and S2 Table”. The eight regions in Butana are located on BTA 4 (five regions), BTA 5 (two regions), and BTA 21 (one region). The two regions observe in Kenana were on BTA 6 and BTA 7. The *Rsb* analyses of Butana cattle show 7, 5,42, 21 and 4 candidate regions across 20 autosomes against European taurine (HOL and JER), NDM, EASZ, SHK and NEL cattle populations, respectively “Fig 4 and S2 Table”. The *Rsb* analyses between Kenana cattle and the five groups of non-Sudanese cattle, identify 6, 8, 26, 14 and 5 candidate regions across 17 autosomes “Fig 5 and S2 Table”.

None of the candidate regions identified by the *iHS* analysis in Butana cattle overlaps with those identified in Kenana cattle. However, 20 regions identified by the *Rsb* analysis overlap between Butana and Kenana populations. These regions include nine from the comparison with EASZ, four with European taurine, one with N’Dama, four with Sheko and two with Nelore “S3 Table”. A total of nine candidate regions identified by the different *Rsb* analyses overlap with the ones identified by the *iHS* analyses, i.e. five in Butana and four in Kenana “S4 Table”.

### Overlap with candidate regions under positive selection in other cattle populations

Four and 28 of the Butana candidate regions, identified by *iHS* and *Rsb* analyses, respectively, have been identified in other studies. Twenty-five of the *Rsb* candidate regions, but none of *iHS* candidate regions, in Kenana cattle have been identified in other studies. These include indigenous tropical-adapted cattle such as Creole [26], Gir [43], West African Borgou [44], EASZ [18] and Ankole [25], and commercial beef and dairy cattle, e.g Holstein [21], Jersey, Angus, Charolais, Hereford and Murray Grey [22, 45] “S2 Table”.

### Identification of genes and QTL within the candidate regions

A total of 366 and 245 genes are found in the candidate regions identified in Butana and Kenana cattle, respectively “S5 and S6 Tables”. The functional annotation analysis conducted on these genes identifies 32 and 14 functional term clusters in Butana and Kenana, respectively “S7 Table”. A total of five and three functional term clusters for Butana and Kenana cattle, respectively, are significantly enriched (enrichment score > 1.3), relative to the bovine genome “Table 1”.

**Table 1:** Significantly enriched functional term clusters and their enrichment scores following DAVID analysis for genes identified in Butana and Kenana candidate regions.

Butana		Kenana	
Functional cluster	Enrichment score*	Functional cluster	Enrichment score*
Olfactory receptor activity	7.37	Peptidase inhibitor activity	2.09
Lipid metabolism	5.25	Olfactory receptor activity	5.12
Peptidase activity	3.16	Serum Ablumin	3.27
Blood coagulation	1.79		
Lipoxygense activity	1.74		

*Enrichment score following DAVID analysis (a score equals to 1.3, equivalent to Fisher exact test *P*-value = 0.05, was used as a significant threshold).

Based on the 25 kb interval distance up- and down-stream of the most significant SNPs in the candidate regions (see materials and methods section), 71 and 51 genes “S8 and S9 Tables” were considered as probable candidates under selection in Butana and Kenana, respectively. Functional annotation analysis shows that these genes are associated with different biological functions, including olfactory receptor activity, component of plasma membrane and kinase activity “S10 Table”. A total of 25 candidate regions (nine in Butana and 16 in Kenana) did not span any genes (i.e. gene deserts) based on the UMD 3.1 reference genome assembly “S11 Table”.

A total of 778 and 702 QTL intersect with the candidate regions identified in Butana and Kenana, respectively “S12 Table”. These QTLs are associated with several biological functions, such as immunity (e.g. tick resistance), reproduction (e.g. ovulation rate and gestation length) and dairy production traits (e.g. milk protein percentage and milk yield). Five of the QTLs controlling trypanotolerance, which were identified in a cross between the trypanotolerant West African N'Dama and the susceptible Kenyan Boran cattle [46], overlapped candidate regions identified in the Kenana cattle “S13 Table”. None of the trypanotolerant reported QTL [46] overlaps with candidate signature of selection in Butana.

## Discussion

### Butana and Kenana genomic structure

In this study, we analyzed two breeds of indigenous East African zebu dairy cattle from Sudan (Butana and Kenana), using genome-wide high density SNP genotype data, to assess their genomic structure and identify possible candidate signatures of positive selection. Based on prior knowledge regarding the origins of cattle breeds (Europe, Africa and Asia), and previous findings of the presence of three genetic backgrounds in cattle breeds [4, 8, 9, 47], we believe that *K* = 3, rather than *K* = 2 as suggested by the Δ*K* approach, represents the optimal number of genetic clusters explaining the greatest variation in the dataset. The latter result (*K* = 2) might be due to the overwhelming effect of the ancient divergence between indicine and taurine cattle relative to the hierarchical relationships between the different cattle populations examined here [8, 48]. Interestingly, fine-scale sub-structure between population are revealed in our study at values of *K* ≥ 4, suggesting a common genetic background unique to the East African cattle population examined here. The origin of this background (e.g. African specific or of Asian origin) remains at this stage speculative.

As already known for other African zebu cattle [8, 9], the PCA and ADMIXTURE analyses revealed the genomes of the Butana and Kenana cattle to be an admixture of indicine and African taurine ancestries. Similar findings were reported previously on these two cattle breeds using autosomal microsatellite markers [49]. It is now well established that the first domestic cattle on the African continent were of taurine type. Humped cattle, in Africa, are of more recent origin following a likely process of male-mediated zebu introgression into African taurine cattle [50] with all African cattle analyzed so far carrying taurine mtDNA haplotypes [5, 7, 51].

The PCA plot on Butana and Kenana cattle indicates a genetic distinction between the Butana from farmers in Tamboul area and the Butana from the Atbara Livestock Research Station, with the former showing higher genetic heterogeneity. This may be explained by higher level of inbreeding in cattle from the research station, where a small number of bulls are used for mating, in comparison to cattle from farmers’ stocks, where random mating with larger number of bulls is usually followed. Moreover, a signature of European taurine introgression was observed in ten individuals of Butana, which were sampled from farmers’ herds in Tamboul area. This may be the outcome of individual farmer past efforts of crossbreeding with European dairy breeds to increase milk production.

### Signatures of selection in Butana and Kenana

We investigated signatures of positive selection in the genomes of Butana and Kenana cattle using two approaches (*iHS* and *Rsb*). A total of 87 and 61 candidate genomic regions under positive selection were identified in Butana and Kenana cattle, respectively. These candidate regions harbor genes, and overlap with QTL, associated with different biological traits, such as milk-production, immunity, thermotolerance, coat color and reproduction.

About 38% (n = 57) of the candidate signatures of selection overlap with candidate regions identified previously in other cattle populations. Moreover, 20 candidate regions overlap between the five *Rsb* comparative analyses, and nine between *Rsb* and *iHS* analyses. These observations support the role of selection pressures, rather than bottlenecks, migration and introgression on these signals [52], considering the different demographic history of the populations examined here. The lower number of regions that overlapped between the *iHS* and *Rsb* analyses can be due to the weakness of the *iHS* approach to detect haplotypes approaching fixation. Moreover, *Rsb* cannot detect signatures of selection if the same region is under selection in the two populations compared [53].

The Butana cattle show a higher number of *iHS* candidate regions (8 regions) than Kenana cattle (2 regions). This might be attributed to the higher heterogeneity and the recent European taurine introgression in Butana cattle, which may lead to the excess of haplotypes under selection with intermediate frequencies.

### Milk production traits

Lactase persistence is present among the people of Africa and in particular within the pastoral communities of Sudan [54–56], where milk represents a major source of nutrition. However, to which extent this might have shaped, through a process of co-evolution, the genetic make-up of some of the indigenous African cattle breeds remains unknown.

A total of 30 candidate regions of positive selection, 15 each in Butana and Kenana, overlap with regions under selection identified in commercial dairy cattle, e.g. Holstein [21] and Jersey [22]. However, none of the well-known genes associated with milk-production, such as *DGAT1*, *GHR* and *ABCG2* [57] are intersecting with any of the candidate regions of positive selection identified in these two indigenous African dairy cattle breeds.

Evidences of selection for dairy production trait in Kenana and Butana requires further investigation. In particular, the following explanation in relation to possible selection for milk production trait in Kenana and Butana following our result may be proposed. Selection pressures may be acting on other genes in the regions that are associated with milk production in Butana and Kenana cattle compared to dairy taurine breed, milk production traits may be under the genetic control of regulatory sequences or genes with pleiotropic effects and/or in linkage disequilibrium with other genes influencing milk production may be under selection in the candidate regions. Further studies are necessary to ascertain this (e.g transcriptome analysis in relation to milk production records [58]).

### Acquired and innate immunity

Due to the widespread presence of infectious and parasitic diseases in the Butana and Kenana areas [12, 13], immunity-related genes and QTL are expected to be a target of selection in these two cattle breeds. Interleukin-1 receptor-associated kinase 3 (*IRAK*) is found in a candidate region on BTA 5 in both Butana and Kenana cattle. This gene plays a role in controlling the inflammation process [59]. Another critical gene within this functional category is interleukin 17B (*IL17B*). This gene, which occurs within a Kenana candidate region on BTA 7, is a member of a cytokine family involved in autoimmunity [60].

Moreover, several immunological-related QTL, such as tick resistance and white blood cell count, are found within the identified candidate regions in Butana and Kenana. The presence of a trypanotolerance QTL overlapping candidate regions for positive selection in Kenana cattle is the first documented evidence that the breed may display some trypanotolerance following environmental pressures, as trypanosomosis is widespread in Sudan [13]. These QTL are associated with different traits, such as decrease in percentage of packed cell volume, parasitaemia and the mean body weight following infection. A degree of trypanotolerance has been reported in other East African cattle breeds (e.g. Orma Boran, Sheko and Mursi cattle) [61–63], this result is therefore not unexpected. The combination of the dairy characteristics of Kenana and its likely trypanotolerance makes it unique so far amongst African indigenous cattle.

### Thermotolerance

Butana and Kenana cattle inhabit a hot environment (mean annual temperature in Atbara is 30°C, Tamboul and Rabak is 28.6°C (http://www.sudan.climatemps.com/) with a long dry season lasting for 6–8 months [12]. Genes eliciting response to adaptation to heat stress may be therefore the targets for selection in the two populations. Heat shock transcription factor family member 5 (*HSF5*) was found in a candidate region in Butana cattle (BTA 19: 9,525,262–9,783,489). Like other HSF family members, *HSF5* binds to DNA elements upstream to heat-inducible genes (e.g. heat shock proteins) to activate their expression [64]. Under thermal stress, these heat-inducible genes maintain protein folding and structures [65, 66].

### Coat color

Butana cattle are characterized by a deep red coat color while Kenana cattle are born with red coats, which turn grey upon maturity [11, 14]. One of the genes identified in a candidate region in Butana cattle (BTA 5: 57,508,578–57,945,083), which might be linked to this phenomenon, is pre-melanosome (*PMEL*). This gene is involved in the synthesis of eumelanin and may therefore regulate coat color [67, 68]. *PMEL* has also been found in a candidate region under selection in the West African Borgou [44] and small East African shorthorn zebu [18] cattle.

### Reproduction and fertility

As for the East African shorthorn zebu and other African zebu cattle previously studied [18, 19, 44], we identified several signatures of selection including genes associated with reproduction and fertility. Steroid 5-alpha reductase 3 (*SRD5A3*) is a gene that is associated with the development of male reproductive system. This gene, which was found in a candidate region in both Butana and Kenana cattle (BTA 6: 72,468,365–72,518,226), is involved in the conversion of testosterone to dihydrotestosterone to maintain prostate and external genitalia differentiation [69]. Alpha-fetoprotein (*AFP*) is another gene that was present in a candidate region (BTA 6: 90,258,976–90,280,522) in Kenana cattle. Experiments using *AFP* knockout mice showed that it plays a role in ovulation [70].

Also, following functional terms clustering analysis, several olfactory receptor genes were significantly enriched in the Butana and Kenana candidate regions relative to the whole genome. Given their expression in human testes and more specifically in mature spermatozoa, olfactory receptor gene families have been linked to reproduction performance [71–73]. These receptors interact with chemo-attractants that are secreted by oocyte-cumulus cell complexes to direct spermatozoa towards the oocyte [73–75]. Moreover, olfactory receptors in male nasal cavity are involved in detecting pheromones released from females during oestrus [76, 77].

### Gene desert regions

The identification of 25 candidate regions in Butana and Kenana cattle with no annotated genes requires further investigations. These regions may carry unannotated regulatory elements, e.g. long non-coding RNA (lnRNA), or coding genes, which can be validated by RNA sequencing analysis.

## Conclusion

By using high density genome-wide SNP genotype data, we reported here the indicine–taurine genomic admixture of two indigenous East African zebu dairy cattle, Butana and Kenana, from Sudan. The genomes of these two cattle breeds are targeted by different selection pressures associated with immunity, thermotolerance and coat color. Our findings open avenues aimed at identifying causative variants that confer adaptation of indigenous cattle to semi-arid environments. Furthermore, our findings may help the designing of foundation breeding programmes to enhance the performance of the Butana and Kenana cattle in their production environments through within-breed selection as well as crossbreeding approaches, in addition to improving management.

## Supporting information

S1 Fig The sampling locations of Butana (Atbara livestock research station and farmers from Tamboul area) and Kenana (Framers from Rabak) cattle. (PDF)

S2 Fig Histograms showing the distribution of the (A) standardized *Rsb* values and (B) standardized *iHS* values.
(TIFF)

S3 Fig Mean r^2^ values over increasing distances across Butana and Kenana autosomes. Values averaged across all the autosomes for each bin size.
(PDF)

S4 Fig The second order rate of change of the likelihood function with respect to the cluster *K* (delta *K*) for ADMIXTURE analysis with K from 1 to 8. (PDF)

S5 Fig ADMIXTURE bar plots of genetic membership proportions for K = 5 to K = 8. Each sample is represented by a vertical line divided into K colours. HOL: Holstein, JER: Jersey, NDM: N’Dama, NEL: Nelore, SHK: Sheko, KEN: Kenana, BUT: Butana.
(GIF)

S1 Table The ID and locations of the genotyped Butana and Kenana cattle samples in this study. (XLSX)

S2 Table Candidate regions identified on Butana and Kenana cattle and the overlaps with previous studies on tropical-adapted cattle and commercial breeds. (XLSX)

S3 Table Overlapping candidate regions between Butana and Kenana *Rsb* analyses. (XLSX)

S4 Table Overlapping candidate regions between *Rsb* and *iHS* analyses. (XLSX)

S5 Table Genes identified within the Butana candidate regions. (XLSX)

S6 Table Genes identified within the Kenana candidate regions. (XLSX)

S7 Table Functional terms enriched clusters on the genes of Butana and Kenana candidate regions. (XLSX)

S8 Table Candidate genes identified within the Butana candidate regions. (XLSX)

S9 Table Candidate genes identified within the Kenana candidate regions. (XLSX)

S10 Table Functional terms enriched clusters on the candidate genes of Butana cattle. (XLSX)

S11 Table Gene desert candidate regions identified in Butana and Kenana cattle. (XLSX)

S12 Table Quantitative trait loci (QTL) overlapping the Butana and Kenana candidate regions. (XLSX)

S13 Table Trypanotolerance QTL, identified by Hanotte et al., (2003), overlapping with Kenana candidate regions. (XLSX)
