# Supplementary figures and images for: Signatures of positive selection in African Butana and Kenana dairy zebu cattle
Source: PLoS One. 2018 Jan 4;13(1):e0190446. doi: 10.1371/journal.pone.0190446 (PMC5754058; doi:10.1371/journal.pone.0190446)

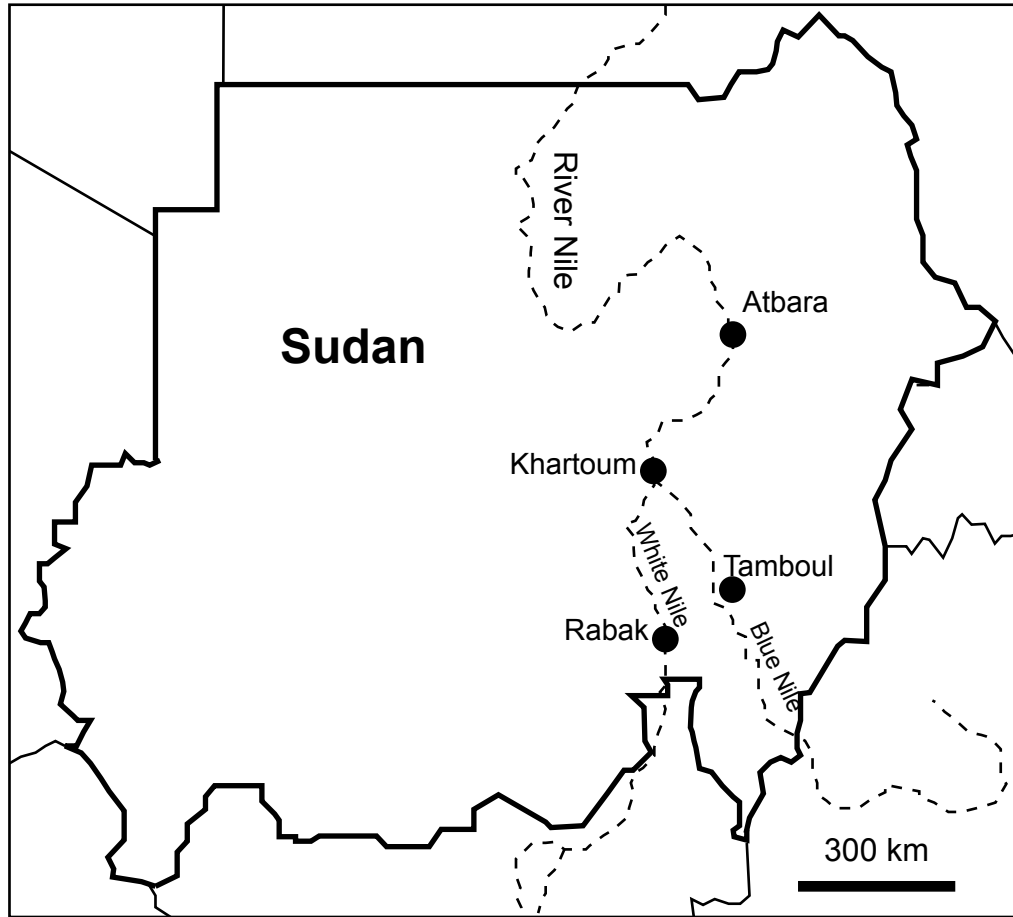

Supplement: S1 Fig — (PDF) [file pone.0190446.s001.pdf]

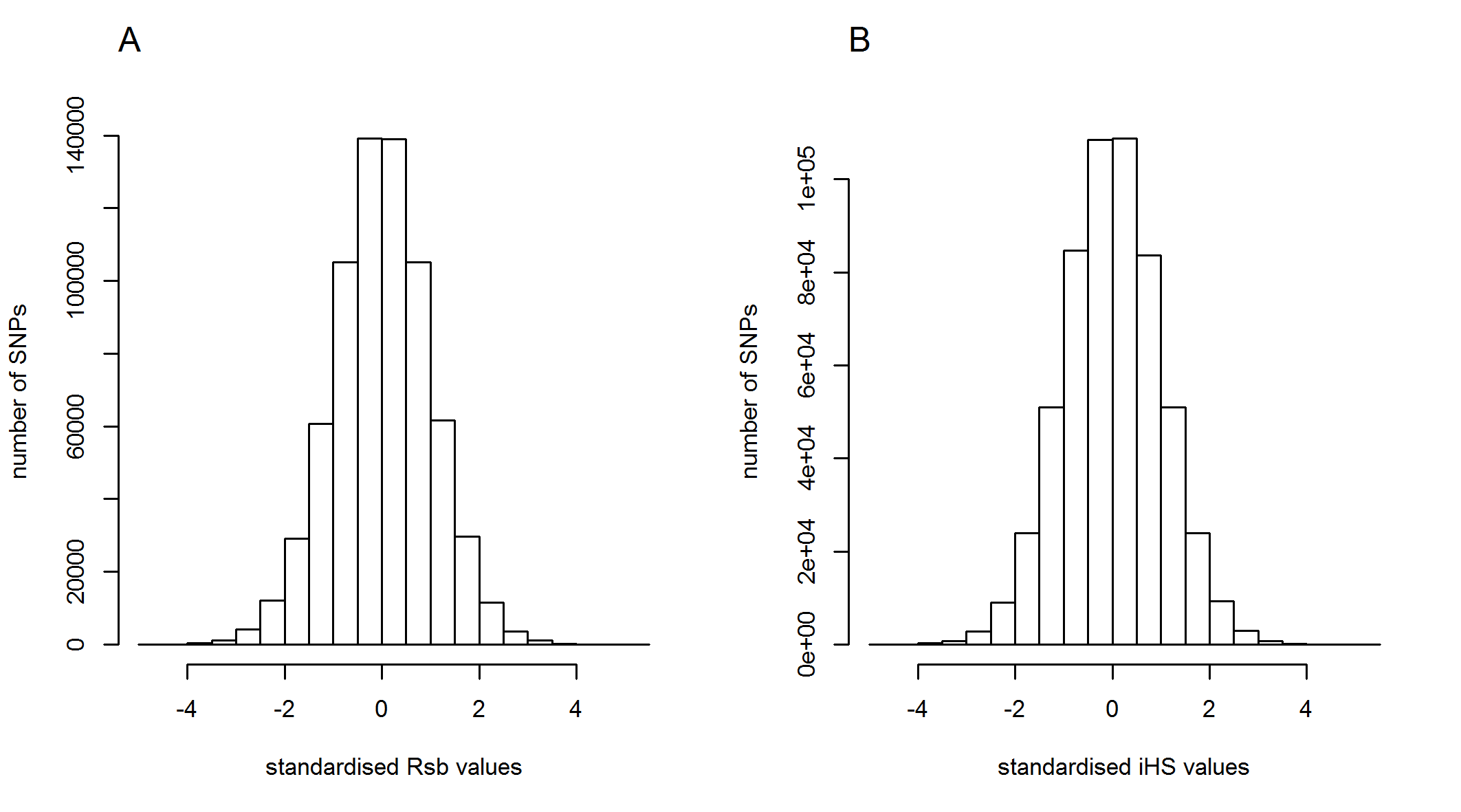

Supplement: S2 Fig — Histograms showing the distribution of the (A) standardized Rsb values and (B) standardized iHS values. (TIFF) [file pone.0190446.s002.tiff]

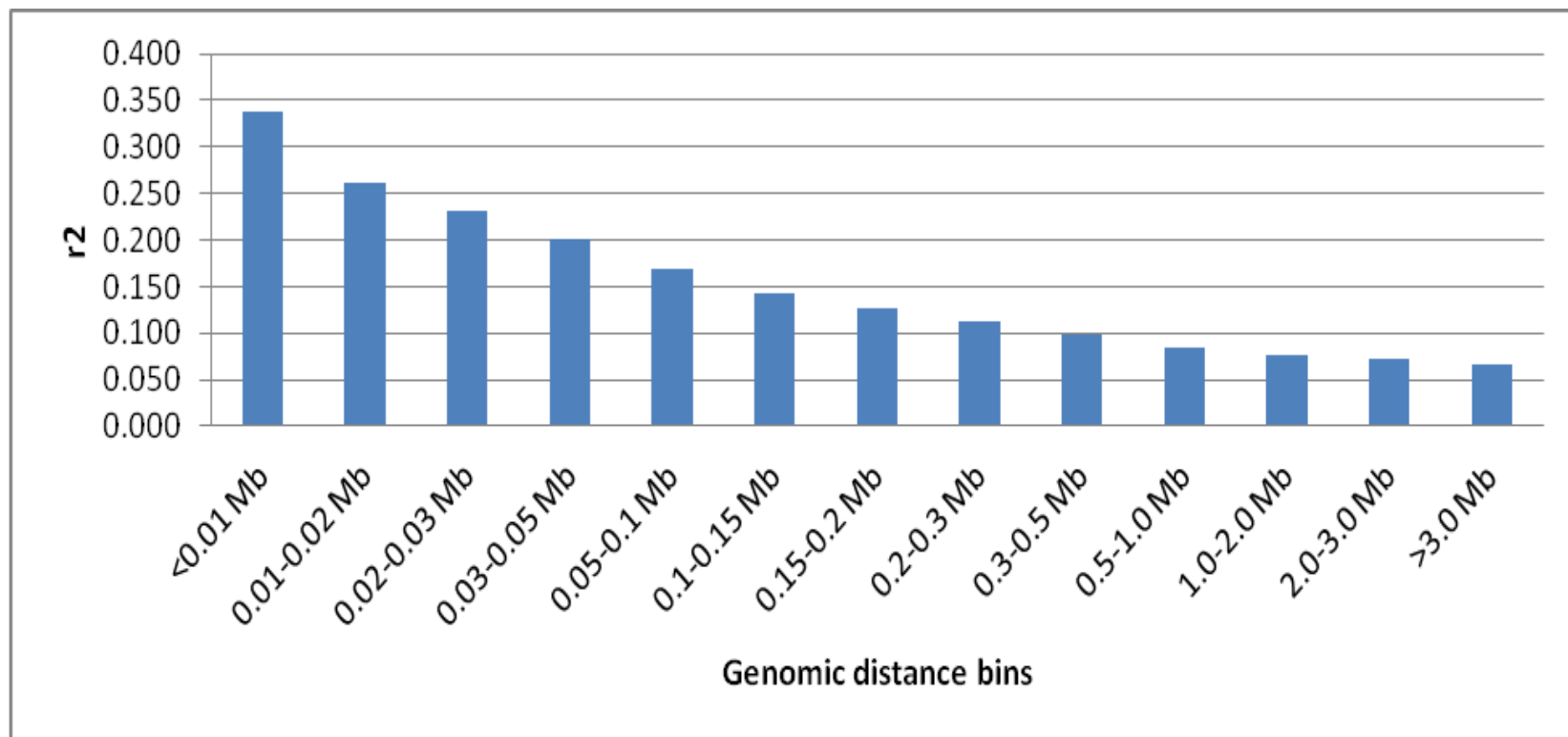

Supplement: S3 Fig — Values averaged across all the autosomes for each bin size. (PDF) [file pone.0190446.s003.pdf]

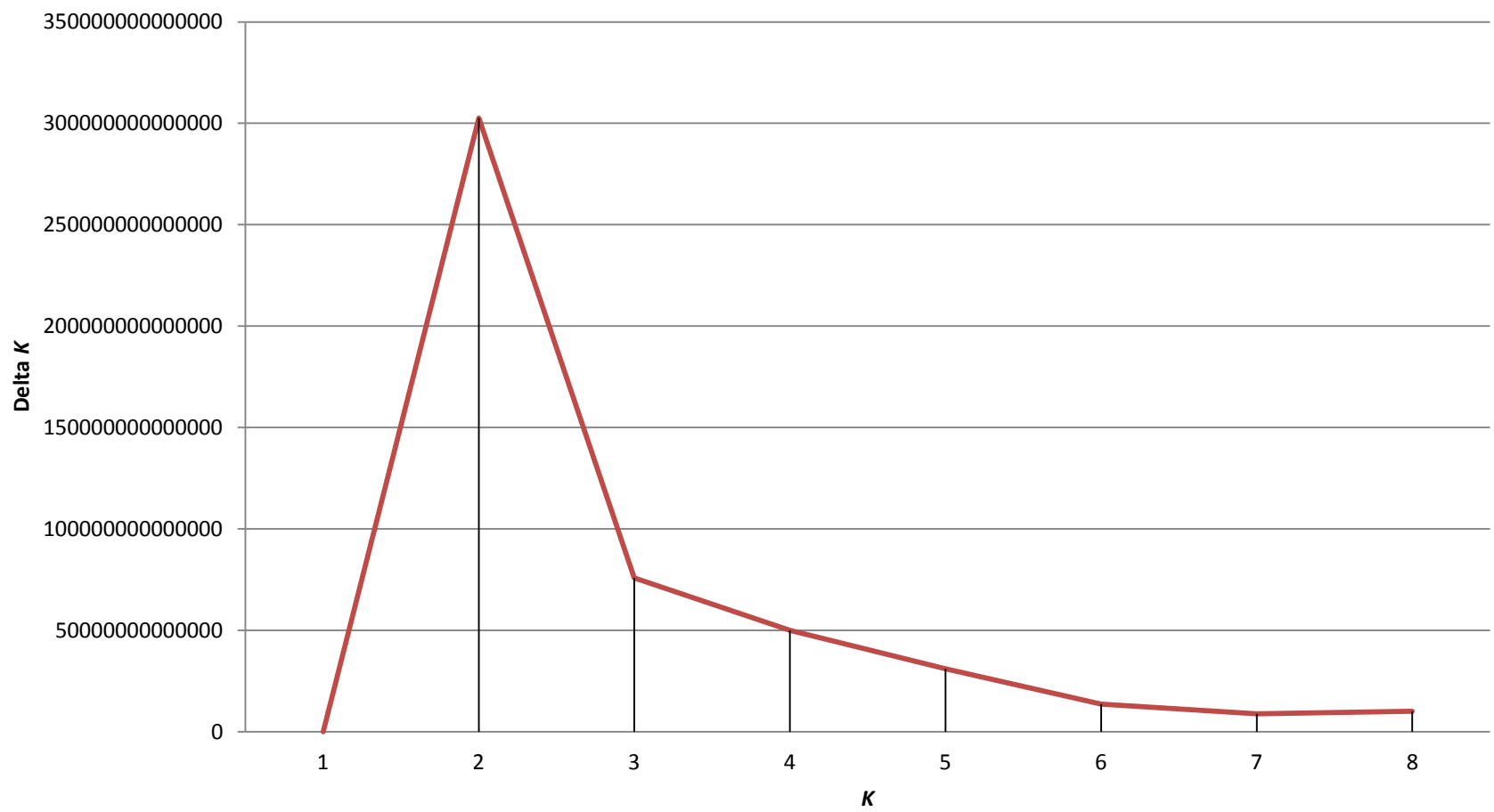

Supplement: S4 Fig — (PDF) [file pone.0190446.s004.pdf]

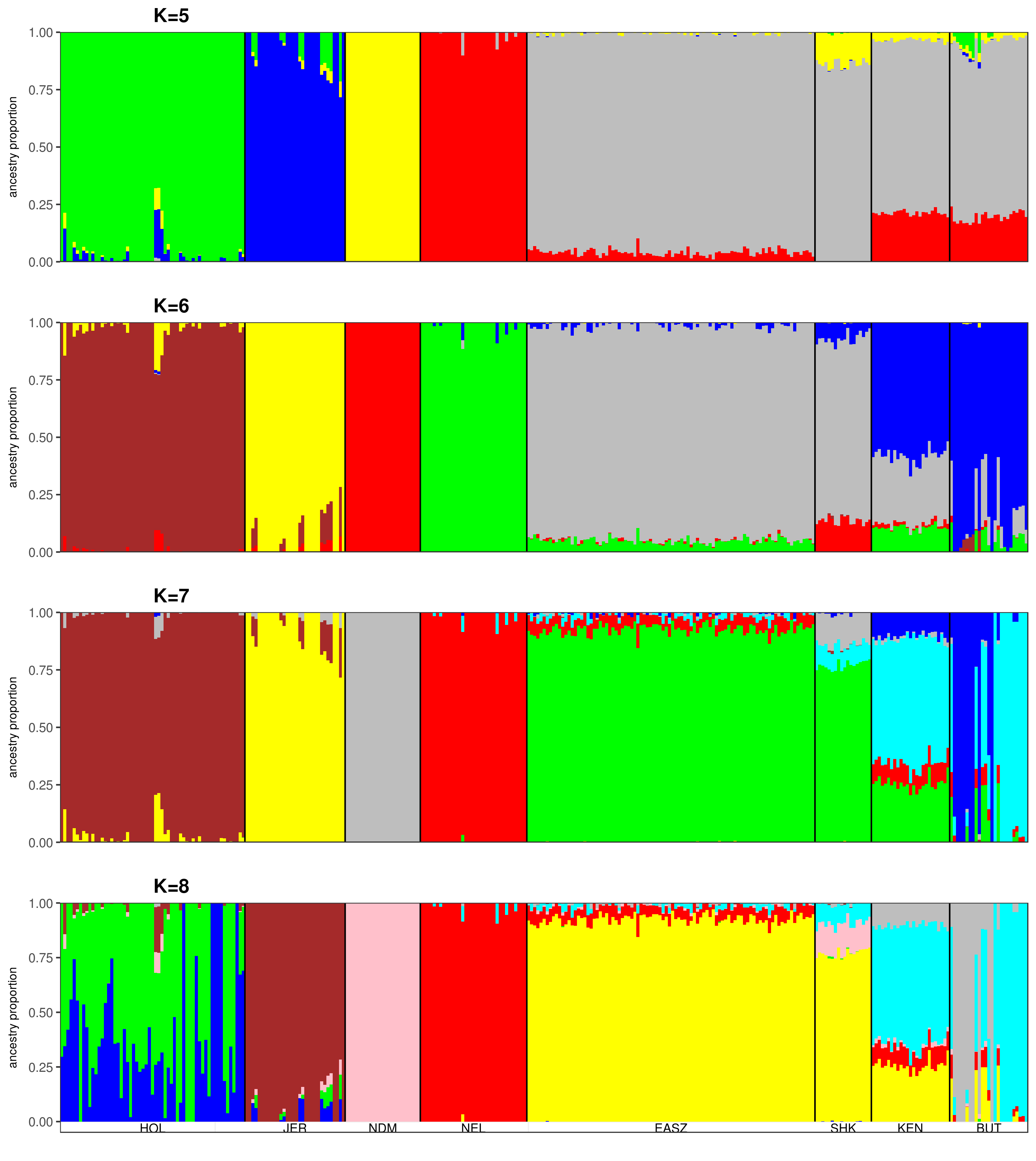

Supplement: S5 Fig — Each sample is represented by a vertical line divided into K colours. HOL: Holstein, JER: Jersey, NDM: N’Dama, NEL: Nelore, SHK: Sheko, KEN: Kenana, BUT: Butana. (GIF) [file pone.0190446.s005.gif]
